# A New Pseudo-Spectral Method Using the Discrete Cosine Transform

**DOI:** 10.3390/jimaging6040015

**Published:** 2020-03-28

**Authors:** Izumi Ito

**Affiliations:** Information and Communications Engineering, Tokyo Institute of Technology, Tokyo 152-8552, Japan; ito@ict.e.titech.ac.jp; Tel.: +81-3-5734-2997

**Keywords:** pseudo-spectral method, derivatives, discrete cosine transform, discrete sine transform

## Abstract

The pseudo-spectral (PS) method on the basis of the Fourier transform is a numerical method for estimating derivatives. Generally, the discrete Fourier transform (DFT) is used when implementing the PS method. However, when the values on both sides of the sequences differ significantly, oscillatory approximations around both sides appear due to the periodicity resulting from the DFT. To address this problem, we propose a new PS method based on symmetric extension. We mathematically derive the proposed method using the discrete cosine transform (DCT) in the forward transform from the relation between DFT and DCT. DCT allows a sequence to function as a symmetrically extended sequence and estimates derivatives in the transformed domain. The superior performance of the proposed method is demonstrated through image interpolation. Potential applications of the proposed method are numerical simulations using the Fourier based PS method in many fields such as fluid dynamics, meteorology, and geophysics.

## 1. Introduction

The discrete cosine transform (DCT) and discrete sine transform (DST) have been extensively studied, and they have played a crucial role in science and engineering for decades. For example, DCT is used for standard image and video compression such as JPEG and MPEG. DST is adopted for high efficiency video coding (HEVC). DCT and DST are closely related to the discrete Fourier transform (DFT) [1,2,3]. All the relevant transforms assume the periodicity of sequences. DFT assumes circular periodicity, where the left side of a sequence is placed next to the right side, while DCT and DST assume symmetric circular periodicity, where after a sequence is extended symmetrically, circular periodicity ensues. There are four types of DCT and DST (Types 1, 2, 3, and 4) corresponding to different types of symmetry. As is well known, DCT Type 2 (DCT-2) is employed for JPEG and MPEG, and DCT Type 3 is the inverse transform of DCT-2. Some applications use several types of DCT and DST, while others just use one type [4,5].

Pseudo-spectral (PS) methods originated from [6,7] and have been studied for solutions of partial differential equations [8]. The numerical solution is obtained via a finite set of expansion functions. Generally, for periodic problems, the Fourier series is used as expansion functions, while for non-periodic problems, orthogonal polynomials (e.g., Jacobi polynomials) are used. Legendre and Chebyshev polynomials are special cases of Jacobi polynomials, in which derivatives are obtained at unequally spaced points, such as Legendre–Gauss–Lobatto points and Chebyshev–Gauss–Lobatto points (also referred to as Chebyshev points) [9,10,11]. Different expansion functions for PS methods are chosen depending on the problems of applications. The PS method using Fourier series as expansion functions is employed for estimating derivatives at equally spaced points, which is common in fluid dynamics [12,13], meteorology, and geophysics, e.g., a direct numerical simulation for a turbulent flow [14], wave prediction [15], multibody modeling of wave energy converters [16], and seismogram simulation [17]. Generally, DFT is used for the PS method, and in periodic functions, approximations by the PS method using DFT (PS-DFT) are much more accurate than those by finite difference methods [18]. Moreover, the use of the fast Fourier transform (FFT) accelerates the process. However, conversely, use of DFT/FFT is problematic due to circular periodicity. If the values on both sides of sequences differ significantly, oscillatory approximations are observed around both sides, which is already well known as the Gibbs phenomenon.

Image interpolation by Hermite polynomials is a good example to understand the accuracy of derivatives. It requires pixel intensities and their derivatives that affect the accuracy of interpolation, where there is room for the choice of methods for calculating derivatives [19,20]. The combination with PS-DFT has been shown to outperform conventional interpolation methods such as bicubic and Lanczos in terms of both accuracy and speed [21]. However, it has not hitherto touched on the adverse effects by PS-DFT.

In the present paper, we study PS methods based on symmetric extension to address the problem of the Gibbs phenomenon induced by PS-DFT in image interpolation. We are motivated by the seminal work [21] combining Hermite polynomials with PS-DFT for image interpolation. We focus on the symmetric extension of a sequence in the spatial domain, which attenuates the difference in values at both sides for suppressing the Gibbs phenomenon. The DCT can enable sequences to function as symmetrical extended sequences. We mathematically derive two types of PS methods using DCT from the relation between DFT and DCT. We evaluate the proposed methods through combining with Hermite polynomials for image interpolation. The results testify to the efficacy of the PS method based on symmetric extension. This paper thus extends earlier results available in the literature [22].

The remainder of the paper is organized as follows. In Section 2, we provide preliminaries in the form of relevant definitions. We present and derive two PS methods based on symmetric extension in Section 3. Our evaluations of image interpolation are detailed in Section 4. Finally, conclusions are put forward in Section 5.

Notations: Let Z and R be the sets of integers and real numbers, respectively. Sequences and signals in the time domain are represented as lower case letters, and their coefficients in the transformed domain are denoted as upper case letters. The operator $T^{-1}$ indicates an inverse transform that assigns a sequence in the transformed domain to a corresponding sequence in the time domain. The derivatives of x(n) with respect to *n* are represented as $x^{\prime }\left(n\right)$. We use an asterisk to denote the complex conjugate, i.e., $X^{*}\left(k\right)$ is the complex conjugate of X(k).

## 2. Preliminaries

First, we provide the definitions of the relevant transforms and their relations. Then, we briefly describe the PS method using DFT.

### 2.1. Definitions of DFT, DCT-1, and DCT-2

Let x(n), n∈Z be a sequence of length *N*. The forward and inverse discrete Fourier transforms are defined as:

(1) $$\begin{array}{c} X\left(k\right)=\sum_{n=0}^{N-1}x\left(n\right)W_{N}^{nk} \end{array}$$

and:

(2) $$\begin{array}{c} x\left(n\right)=\frac{1}{N}\sum_{k=0}^{N-1}X\left(k\right)W_{N}^{-nk} \end{array}$$

respectively, where $W_{N}=\exp\left(-j2\pi /N\right)$ and $j=\sqrt{-1}$.

DCT Type 1 (DCT-1) is defined [23] as:

(3) $$\begin{array}{c} X_{C_{1}}\left(k\right)=2\sum_{n=0}^{N-1}\alpha \left(n\right)x\left(n\right)\cos\left(\frac{\pi kn}{N-1}\right) \end{array}$$

where:

(4) $$\begin{array}{cc} \alpha (n) & =\left\{\begin{array}{cc} 1/2, & n=0,N-1\\ 1, & \mathrm{otherwise} \end{array}\right.. \end{array}$$

DCT Type 2 (DCT-2) is defined as:

(5) $$\begin{array}{c} X_{C_{2}}\left(k\right)=\sqrt{\frac{2}{N}}\beta \left(k\right)\sum_{n=0}^{N-1}x\left(n\right)\cos\left(\frac{\pi k(n+1/2)}{N}\right) \end{array}$$

where:

(6) $$\begin{array}{cc} \beta (k) & =\left\{\begin{array}{cc} 1/\sqrt{2}, & k=0\\ 1, & \mathrm{otherwise} \end{array}\right.. \end{array}$$

Note that the inverse DCT-1 corresponds to DCT-1 and that the inverse DCT-2 corresponds to DCT Type 3.

### 2.2. Relation between DFT and DCT

**Figure 1:**
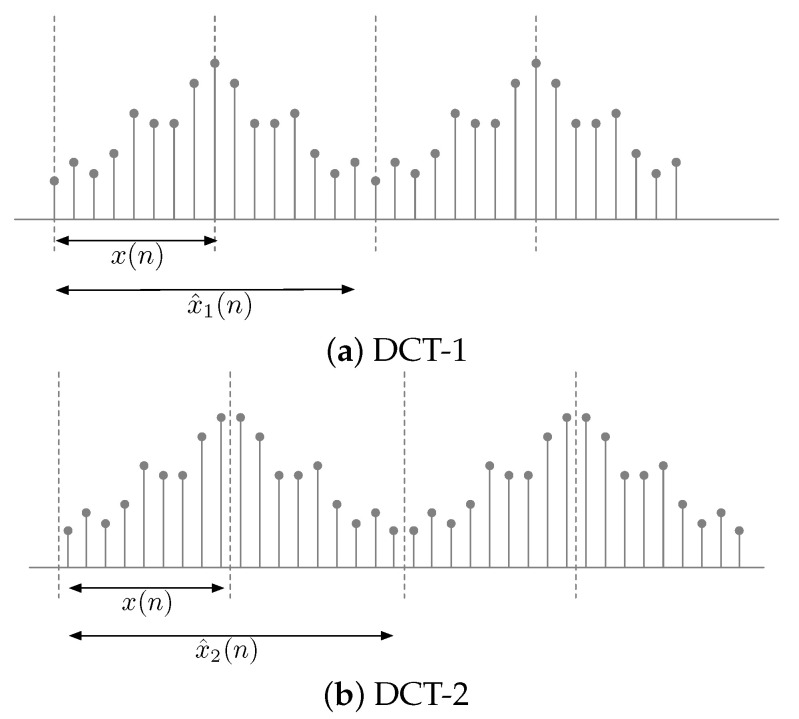
Original sequence and symmetrically extended sequence.

The DCT coefficients of an original sequence correspond to the DFT coefficients of the sequence to which the original sequence is extended symmetrically. Let x(n) be the original sequence of length *N*. Figure 1 shows an original sequence and the sequence extended symmetrically depending on the type of DCT. The difference between DCT-1 and DCT-2 is where the axis of symmetry lies. Specifically, the axis of symmetry of DCT-1 lies directly between the samples of a sequence, while that of DCT-2 lies at a half-sample between the samples.

The symmetrically extended sequence, ${\hat{x}}_{1}\left(n\right)$, for DCT-1 from x(n), as shown in Figure 1a, is given as:

(7) $$\begin{array}{c} {\hat{x}}_{1}\left(n\right)=\left\{\begin{array}{cc} x(n) & n=0,1,\cdots ,N-1\\ x(2N-2-n) & n=N,N+1,\cdots ,2N-3 \end{array}\right.. \end{array}$$

The relation between DFT coefficients and DCT-1 coefficients is expressed for k=0,1,⋯,N−1 as:

(8) $$\begin{array}{c} {\hat{X}}_{1}\left(k\right)=X_{C_{1}}\left(k\right) \end{array}$$

where ${\hat{X}}_{1}\left(k\right)$ is the DFT coefficients of ${\hat{x}}_{1}\left(n\right)$ and $X_{C_{1}}\left(k\right)$ is the DCT-1 coefficients of x(n).

The symmetrically extended sequence, ${\hat{x}}_{2}\left(n\right)$, for DCT-2, as shown in Figure 1b, is given from x(n) as:

(9) $$\begin{array}{c} {\hat{x}}_{2}\left(n\right)=\left\{\begin{array}{cc} x(n) & n=0,1,\cdots ,N-1\\ x(2N-n-1) & n=N,N+1,\cdots ,2N-1 \end{array}\right.. \end{array}$$

The relation between DFT coefficients and DCT-2 coefficients is expressed for k=0,1,⋯,N−1 as:

(10) $$\begin{array}{c} {\hat{X}}_{2}\left(k\right)=\sqrt{2N}\frac{1}{\alpha (k)}X_{C_{2}}\left(k\right)W_{2N}^{-k/2} \end{array}$$

where ${\hat{X}}_{2}\left(k\right)$ is the DFT coefficients of ${\hat{x}}_{2}\left(n\right)$ and $X_{C_{2}}\left(k\right)$ is the DCT-2 coefficients of x(n).

Thus, DCTs can enable sequences to behave as symmetrically extended sequences without the manual extension of symmetry.

### 2.3. PS Method Using the DFT

The PS method using DFT (PS-DFT) is a numerical method for estimating the derivatives of sequences. The underlying theory behind PS-DFT is the time derivative properties of the Fourier transform.

We assume x(n) are samples of a differentiable continuous-time signal, $x_{a}\left(t\right)$, t∈R at time $nT_{s}$, i.e., $x\left(n\right)=x_{a}\left(nT_{s}\right)$ where $T_{s}$ represents a sampling period that satisfies the Nyquist sampling theorem. When the time axis is normalized by a factor of $T_{s}$, the samples of the derivative of $x_{a}\left(t\right)$ with respect to *t* are written as:

(11) $$\begin{array}{c} \frac{\partial }{\partial t}x_{a}\left(nT_{s}\right)=\frac{\partial }{\partial n}x\left(n\right) \end{array}$$

where 1=∂n/∂t from $t=nT_{s}$ and $T_{s}=1$.

From (2), the derivative, $x^{\prime }\left(n\right)$, of x(n) with respect to *n* is expressed as:

(12) $$\begin{array}{c} x^{\prime }\left(n\right)=\frac{\partial }{\partial n}x\left(n\right)=\frac{1}{N}\sum_{k=0}^{N-1}\frac{j2\pi k}{N}X\left(k\right)W_{N}^{-nk}. \end{array}$$

Observe that the DFT coefficient, $X^{\prime }\left(k\right)$, of $x^{\prime }\left(n\right)$ is given in (12) as:

(13) $$\begin{array}{c} X^{\prime }\left(k\right)=\frac{j2\pi k}{N}X\left(k\right). \end{array}$$

Note that the coefficients $X^{\prime }\left(k\right)$ require complex conjugate symmetry so that $x^{\prime }\left(n\right)$ are real numbers.

Therefore, we can obtain the derivatives by the inverse DFT from the coefficients multiplying the DFT coefficients of the original sequence by the factors with respect to *k*. The steps involved in implementing PS-DFT are summarized as follows.

1. *N*-point DFT is applied to the sequence of length *N* to obtain X(k) according to (1).
2. X(k) is multiplied by j2πk/N to obtain $X^{\prime }\left(k\right)$ according to (13).
3. The inverse DFT is applied to $X^{\prime }\left(k\right)$ according to (2).

## 3. PS Method Based on Symmetric Extension

Here, we derive two PS methods depending on the type of symmetry extension, considering the PS-DFT of symmetrically extended sequences. We assume that the symmetrically extended sequence is the samples that satisfy the Nyquist sampling theorem of a differentiable continuous-time signal where the time axis is normalized.

### 3.1. Derivation of PS-DCT1

Let us consider the derivative, ${\hat{x}}_{1}^{\prime }\left(n\right)$, of the symmetrically extended sequence, ${\hat{x}}_{1}\left(n\right)$, for DCT-1 in (7).

From (2), ${\hat{x}}_{1}^{\prime }\left(n\right)$ is given as:

(14) $$\begin{array}{cc} {\hat{x}}_{1}^{\prime }\left(n\right)=\frac{\partial }{\partial n}{\hat{x}}_{1}\left(n\right) & =\frac{\partial }{\partial n}\left(\frac{1}{2M}\sum_{k=0}^{2M-1}{\hat{X}}_{1}\left(k\right)W_{2M}^{-nk}\right) \end{array}$$

where M=N−1. With the symmetry properties of DFT, it is developed as:

(15) $$\begin{array}{cc} {\hat{x}}_{1}^{\prime }\left(n\right) & =\frac{1}{2M}\frac{\partial }{\partial n}\left(\sum_{k=0}^{M-1}{\hat{X}}_{1}\left(k\right)W_{2M}^{-nk}+\sum_{k=M}^{2M-1}{\hat{X}}_{1}\left(k\right)W_{2M}^{-nk}\right) \end{array}$$

(16) $$\begin{array}{cc} \; & =\frac{1}{2M}\frac{\partial }{\partial n}\left(\sum_{k=1}^{M-1}{\hat{X}}_{1}\left(k\right)W_{2M}^{-nk}+\sum_{k=1}^{M-1}{\hat{X}}_{1}^{*}\left(k\right)W_{2M}^{nk}\right) \end{array}$$

(17) $$\begin{array}{cc} \; & =\frac{1}{2M}\left(\sum_{k=1}^{M-1}\frac{j2\pi k}{2M}{\hat{X}}_{1}\left(k\right)W_{2M}^{-nk}-\sum_{k=1}^{M-1}\frac{j2\pi k}{2M}{\hat{X}}_{1}^{*}\left(k\right)W_{2M}^{nk}\right) \end{array}$$

From (8), replacing ${\hat{X}}_{1}\left(k\right)$ by $X_{C_{1}}\left(k\right)$, we have:

(18) $$\begin{array}{cc} {\hat{x}}_{1}^{\prime }\left(n\right) & =\frac{1}{2M}\left(\sum_{k=1}^{M-1}\frac{j\pi k}{M}X_{C_{1}}\left(k\right)\left(W_{2M}^{-nk}-W_{2M}^{nk}\right)\right) \end{array}$$

(19) $$\begin{array}{cc} \; & =-\frac{1}{M}\sum_{k=1}^{M-1}\frac{\pi k}{M}X_{C_{1}}\left(k\right)\sin\left(\frac{\pi nk}{M}\right). \end{array}$$

Therefore, we define the PS method using DCT-1 in the forward transform as PS-DCT1 together with the inverse transform, $T_{PS-DCT1}^{-1}$, given by:

(20) $$\begin{array}{c} {\hat{x^{\prime }}}_{1}\left(n\right)=T_{PS-DCT1}^{-1}\left[X_{C_{1}}^{\prime }\left(k\right)\right]=-\frac{1}{N\;-\;1}\sum_{k=1}^{N-2}X_{C_{1}}^{\prime }\left(k\right)\sin\left(\frac{\pi nk}{N\;-\;1}\right) \end{array}$$

where:

(21) $$\begin{array}{c} X_{C_{1}}^{\prime }\left(k\right)=\frac{\pi k}{N-1}X_{C_{1}}\left(k\right). \end{array}$$

Note that $T_{PS-DCT1}^{-1}$ corresponds to a sign inverted DST Type 1 (DST-1).

### 3.2. Derivation of PS-DCT2

In a similar manner, PS-DCT2 is derived. From the definition in (2), the derivative, ${\hat{x}}_{2}^{\prime }\left(n\right)$, of the symmetrically extended sequence, ${\hat{x}}_{2}\left(n\right)$, for DCT-2 in (9) is expressed with the DFT coefficients, ${\hat{X}}_{2}\left(n\right)$, of ${\hat{x}}_{2}\left(n\right)$ as:

(22) $$\begin{array}{c} {\hat{x}}_{2}^{\prime }\left(n\right)=\frac{\partial }{\partial n}{\hat{x}}_{2}\left(n\right)=\frac{\partial }{\partial n}\left(\frac{1}{2N}\sum_{k=0}^{2N-1}{\hat{X}}_{2}\left(k\right)W_{2N}^{-nk}\right) \end{array}$$

which is developed with the symmetry properties of DFT as:

(23) $$\begin{array}{cc} {\hat{x}}_{2}^{\prime }\left(n\right) & =\frac{1}{2N}\frac{\partial }{\partial n}\left(\sum_{k=0}^{N-1}{\hat{X}}_{2}\left(k\right)W_{2N}^{-nk}+\sum_{k=N}^{2N-1}{\hat{X}}_{2}\left(k\right)W_{2N}^{-nk}\right) \end{array}$$

(24) $$\begin{array}{cc} \; & =\frac{1}{2N}\frac{\partial }{\partial n}\left(\sum_{k=0}^{N-1}{\hat{X}}_{2}\left(k\right)W_{2N}^{-nk}+\sum_{k=0}^{N-1}{\hat{X}}_{2}^{*}\left(k\right)W_{2N}^{nk}\right) \end{array}$$

(25) $$\begin{array}{cc} \; & =\frac{1}{2N}\left(\sum_{k=0}^{N-1}\frac{j\pi k}{N}{\hat{X}}_{2}\left(k\right)W_{2N}^{-nk}-\sum_{k=0}^{N-1}\frac{j\pi k}{N}{\hat{X}}_{2}^{*}\left(k\right)W_{2N}^{nk}\right) \end{array}$$

From (10), we have:

(26) $$\begin{array}{cc} {\hat{x}}_{2}^{\prime }\left(n\right) & =\frac{1}{\sqrt{2N}}\sum_{k=0}^{N-1}\left(\frac{j\pi k}{N}X_{C_{2}}\left(k\right)W_{2N}^{-\frac{k}{2}}W_{2N}^{-kn}-\frac{j\pi k}{N}X_{C_{2}}\left(k\right)W_{2N}^{\frac{k}{2}}W_{2N}^{kn}\right) \end{array}$$

(27) $$\begin{array}{cc} \; & =-\sqrt{\frac{2}{N}}\sum_{k=0}^{N-1}\frac{\pi k}{N}X_{C_{2}}\left(k\right)\sin\left(\frac{\pi k(n+1/2)}{N}\right). \end{array}$$

Therefore, we define the PS method using DCT-2 in the forward transform as PS-DCT2 together with the inverse transform, $T_{PS-DCT2}^{-1}$, given by:

(28) $$\begin{array}{c} {\hat{x^{\prime }}}_{2}\left(n\right)=T_{PS-DCT2}^{-1}\left[X_{C_{2}}^{\prime }\left(k\right)\right]=-\sqrt{\frac{2}{N}}\sum_{k=0}^{N-1}X_{C_{2}}^{\prime }\left(k\right)\sin\left(\frac{\pi k(n+1/2)}{N}\right) \end{array}$$

where:

(29) $$\begin{array}{c} X_{C_{2}}^{\prime }\left(k\right)=\frac{\pi k}{N}X_{C_{2}}\left(k\right). \end{array}$$

Note that $T_{PS-DCT2}^{-1}$ corresponds to a sign inverted DST Type 2 (DST-2).

### 3.3. Implementing PS-DCT1/PS-DCT2

The steps involved in implementing PS-DCT1/PS-DCT2 are summarized as follows.

1. DCT-1/DCT-2 is applied to a sequence of length *N* to obtain $X_{C_{1}}\left(k\right)$/$X_{C_{2}}\left(k\right)$ according to (3)/(5).
2. $X_{C_{1}}\left(k\right)$/$X_{C_{2}}\left(k\right)$ is multiplied by a factor, πk/(N−1) or πk/N, to obtain $X_{C_{1}}^{\prime }\left(k\right)$/$X_{C_{2}}^{\prime }\left(k\right)$ according to (21)/(29).
3. $T_{PS-DCT1}^{-1}$ / $T_{PS-DCT2}^{-1}$ is applied to $X_{C_{1}}^{\prime }\left(k\right)$/$X_{C_{2}}^{\prime }\left(k\right)$ according to (20)/(28).

### 3.4. Extension to Second and Higher Derivatives

The second and higher derivatives are obtained by differentiating the first derivative repeatedly. For example, from (28), the second derivatives based on the symmetric extension for DCT-2 are given as:

(30) $$\begin{array}{c} \frac{\partial }{\partial n}{\hat{x^{\prime }}}_{2}\left(n\right)=-\sqrt{\frac{2}{N}}\sum_{k=0}^{N-1}X_{C_{2}}^{\prime \prime }\left(k\right)\cos\left(\frac{\pi k(n+1/2)}{N}\right) \end{array}$$

where:

(31) $$\begin{array}{c} X_{C_{2}}^{\prime \prime }\left(k\right)={\left(\frac{\pi k}{N}\right)}^{2}X_{C_{2}}\left(k\right). \end{array}$$

## 4. Application to Image Interpolation

We focus on whole-image interpolation for applications such as image registration and motion correction, rather than interpolation for a small portion of an image. We evaluate the proposed methods combined with Hermite polynomials for image interpolation in terms of accuracy and speed. Interpolation by Hermite polynomials is referred to as Hermite interpolation.

### 4.1. Hermite Interpolation

Let $y_{i}$ and $y_{i}^{\prime }$ be the nodal value and the nodal derivative at $t_{i}\in Z$, (i=0,1,⋯,I), respectively. The Hermite polynomial f(t), t∈R, of degree 2I+1, is given [19] by:

(32) $$\begin{array}{c} f\left(t\right)=\sum_{i=0}^{I}y_{i}a_{i}\left(t\right)+\sum_{i=0}^{I}y_{i}^{\prime }b_{i}\left(t\right) \end{array}$$

where:

(33) $$\begin{array}{cc} a_{i}\left(t\right) & =\left(1-\left(t-t_{i}\right)\frac{p_{i}^{\prime }\left(t_{i}\right)}{p_{i}\left(t_{i}\right)}\right)\frac{p_{i}\left(t\right)}{p_{i}\left(t_{i}\right)},\;b_{i}\left(t\right)=\left(t-t_{i}\right)\frac{p_{i}\left(t\right)}{p_{i}\left(t_{i}\right)}, \end{array}$$

(34) $$\begin{array}{cc} p_{i}\left(t\right) & ={\left(t-t_{0}\right)}^{2}{\left(t-t_{1}\right)}^{2}\cdots {\left(t-t_{i-1}\right)}^{2}{\left(t-t_{i+1}\right)}^{2}\cdots {\left(t-t_{I}\right)}^{2}. \end{array}$$

when I=1 (degree=3), f(t) in (32) requires two nodal values and two nodal derivatives nearest the location *t* for estimating the value at *t*, which is referred to as cubic Hermite interpolation (CHI).

### 4.2. Methods and Environment

We performed geometrical transformation by interpolation to evaluate the proposed methods. The derivatives required for CHI are obtained by PS-DCT1, PS-DCT2, PS-DFT, first-order forward difference (1-FD), second-order central finite difference (2-CLFD), fourth-order central finite difference (4-CLFD), and fourth-order compact finite difference (4-CTFD) [24]. In addition to these results, we provide results according to the cubic natural spline (CNS) to facilitate comparison with other interpolation methods.

We used a total of 12 grayscale images of size 256×256 in standard image data base (SIDBA) [25]. The resulting images are evaluated by the signal-to-noise ratio (SNR) given as:

(35) $$\begin{array}{c} SNR=10log_{10}\left(\sum_{n}g{\left(n\right)}^{2}/\sum_{n}{\left(r\left(n\right)-g\left(n\right)\right)}^{2}\right) \end{array}$$

where g(n) and r(n) represent the ground truth sample and the final resulting sample, respectively. All algorithms were implemented in MATLAB and run on a macOS system with a 2.3 GHz Intel Core i5 and 8 GB memory.

### 4.3. Image Translation

The image was shifted by 0.05 pixels in the horizontal direction in each step. The output of the previous step was used for the input. After 20 steps, the image was shifted by one pixel.

**Figure 2:**
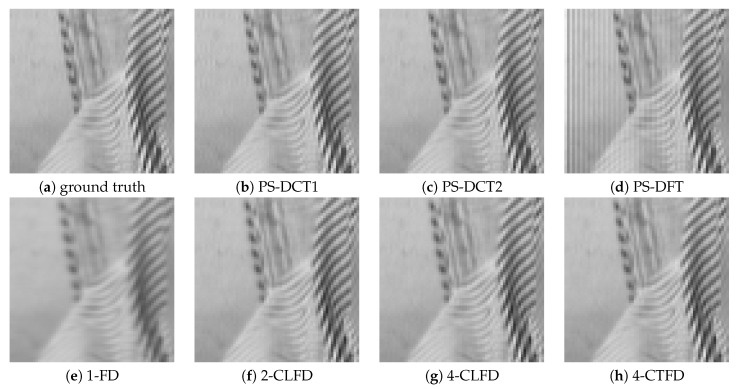
A portion on the leftmost side of the final output after successive image translation of the image “Barbara”. PS, pseudo-spectral; FD, forward difference; CLFD, central finite difference; CTFD, compact finite difference.

**Figure 3:**
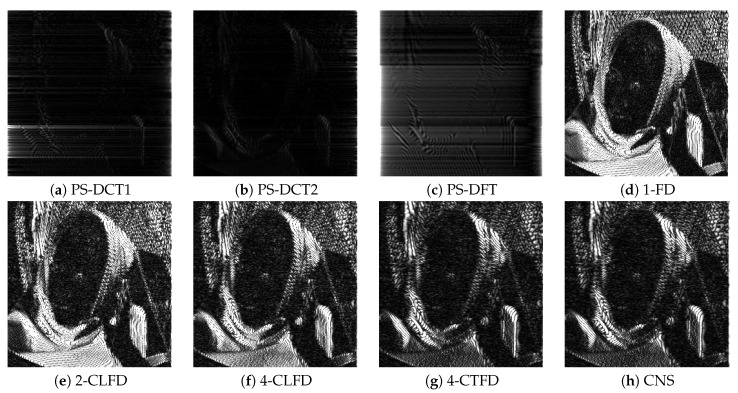
The absolute errors of the final output after successive image translation of the image “Barbara”. CNS, cubic natural spline.

Figure 2 shows a portion on the leftmost side of the final output after successive image translation of the image “Barbara”. The results from PS-DFT suffered severe artifacts compared to those from the other methods. Figure 3 shows the absolute errors of the final output, where black corresponds to zero error, values greater than 20 are white, and values between zero and 20 are intermediate shades of gray. It can be clearly discerned that there is less error associated with PS-DCT1 and PS-DCT2 compared to the other methods. The errors resulting from PS-DCT1, PS-DCT2, and PS-DFT are concentrated on the both sides of the image. Table 1 summarizes the SNRs between the final output and the image shifted by one pixel (ground truth). The highest SNR for each image is in bold. It can be observed that the SNRs of the results from PS-DCT1 and PS-DCT2 were greater than the SNRs of PS-DFT. Accordingly, it was discerned that symmetric extension in the PS method was effective. On average, CNS yielded the highest SNR. However, in several images, PS-DCT2 was better than CNS.

**Figure 4:**
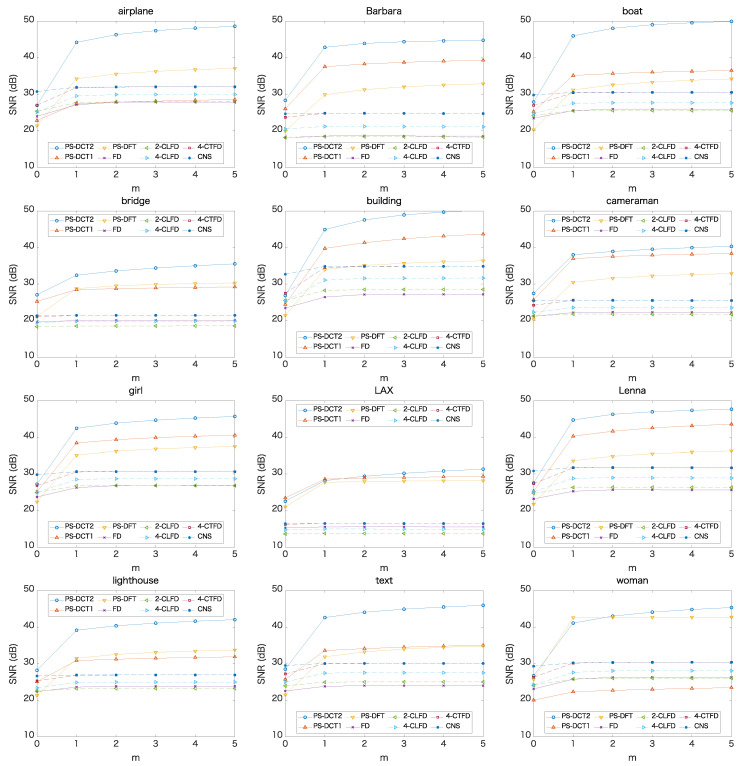
SNRs of the final output when excluding *m* columns on both sides of the image.

Next, we changed the evaluation area. Figure 4 shows the SNRs for each image when the evaluated area was narrowed by *m* columns on each side of the image. It can be seen that the SNRs of PS-DCT1, PS-DCT2, and PS-DFT increased significantly when m=1 and then continued to increase slowly for m>1, while the SNRs of the other methods slightly increased when m=1, then plateaued for m>1. The results imply that there are large adverse effects by PS-DCT1 and PS-DCT2 within one column on both sides of the results. That is, we can obtain superior results by excluding only one pixel at each side. Table 2 summarizes the SNRs of the final output when m=1. Compared to Table 1, the SNRs increased across all methods. Notably, the SNRs of PS-DCT1, PS-DCT2, and PS-DFT increased significantly. PS-DCT2 performed best, except with respect to the image “LAX”.

### 4.4. Image Rotation

The image was rotated by 24 degrees in each step. The output of the previous step was used for the input. After 15 steps, the image was fully rotated. We made each output image the same size as the input, cropping and zero-padding the rotated image to fit. As a result, the part with pixel intensities other than zero of the final output became circular.

**Figure 5:**
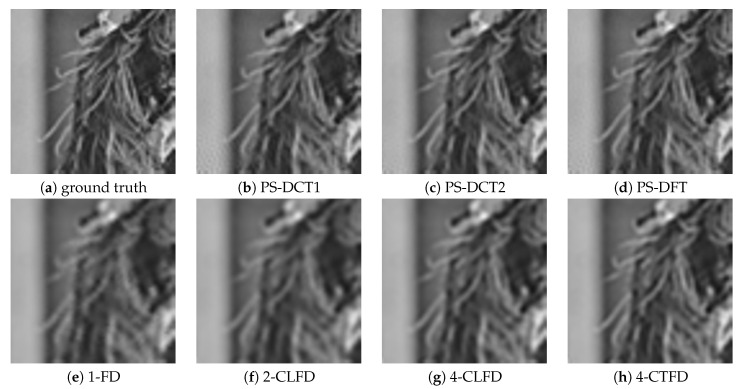
A portion near the circumference of the final output after successive rotation of the image “Lenna”.

**Figure 6:**
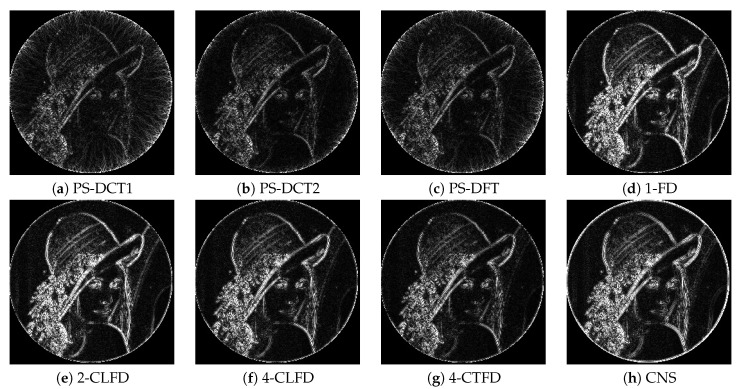
The absolute errors of the final output after successive rotation of the image “Lenna”.

Figure 5 shows a portion near the circumference of the final output after successive rotation of the image “Lenna”. The results from PS-DCT1, PS-DCT2, and PS-DFT were clearer than those from the other methods, but there were minor artifacts in the homogeneous area of the results. Figure 6 shows the absolute errors of the final output. Again, black denotes zero error; values greater than 20 are white; and values between zero and 20 are intermediate shades of gray. There was less error associated with PS-DCT1, PS-DCT2, and PS-DFT compared to the other methods.

Table 3 summarizes the SNRs of the final output when the evaluation area was narrowed by one pixel of the inner the circle, where the highest SNR for each image is in bold. It can be seen that PS-DCT2 yielded the best SNRs of all the methods. However, compared to Table 2, there were no significant differences among PS-DCT1, PS-DCT2, and PS-DFT. One reason for this was that the rotated image for the next input in successive rotation was zero-padded so that the image should be square, which attenuated the Gibbs phenomenon. Another reason was that errors were not concentrated in one place, due to rotation.

### 4.5. Computational Complexity

**Figure 7:**
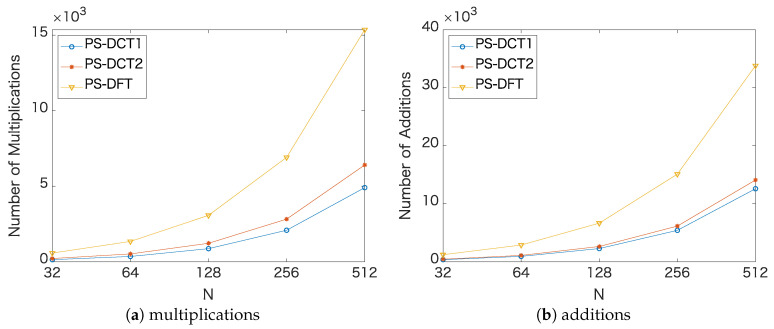
Comparison of the arithmetic operations based on Table 4.

PS-DCT1 requires an *N*-point DCT-1 and an *N*-point DST-1 for the forward and inverse transforms, respectively, and *N* multiplications for the derivatives in the transformed domain. Likewise, PS-DCT2 needs an *N*-point DCT-2, an *N*-point DST-2, and *N* multiplications. PS-DFT requires an *N*-point DFT, an *N*-point inverse DFT, and *N* multiplications. Table 4 summarizes the arithmetic operations of PS-DCT1, PS-DCT2, and PS-DFT for a sequence of length *N*. The operations of PS-DCT1 and PS-DCT2 were based on Wang’s algorithm [26]. The operations of PS-DFT were based on the FFT [27], which were converted to operations in real numbers in which one multiplication of complex numbers was converted to three multiplications and three additions according to Nakayama’s method [28] and one addition of complex numbers was converted to two additions. Figure 7 shows the comparison of the arithmetic operations based on Table 4.

We measured the execution time for image interpolation. All algorithms were executed on a macOS system with a 2.3GHz Intel Core i5 and 8GB memory. A total of 12 images of size 256×256 were used. Table 5 summarizes the mean execution time for successive image translation and successive image rotation. The proposed methods ran slightly more slowly than PS-DFT, but the execution time was acceptable. Together with the above qualities, the results testified to the efficacy of the proposed methods.

## 5. Conclusions

We proposed PS methods based on symmetric extension to attenuate the oscillatory approximation that occurs with DFT. Using DCT, derivatives can be estimated in the transformed domain with sequences behaving as symmetrically extended sequences without a manual extension of symmetry. We derived two PS methods, PS-DCT1 and PS-DCT2, from the relation between DFT and DCT. Through image interpolation by Hermite polynomials, we showed that the proposed methods outperform PS-DFT. DCT can be calculated in real numbers rather than in the complex numbers that are required for DFT. Moreover, DCT has fast algorithms, as well as DFT. Therefore, the proposed methods are advantageous in terms of accuracy and validity compared to PS-DFT.

The potential applications of the proposed method are numerical simulations/modeling using the Fourier based PS method to solve several equations in many fields such as fluid dynamics, meteorology, and geophysics. Furthermore, the proposed method may be applicable to numerical methods where the Fourier based PS method has not been used before, such as those relevant to sensor networks and radar imaging [29,30,31]. The future work of this paper will study potential applications with numerical analysis.

## Figures and Tables

**Table 1:** SNRs (dB) of the final output after successive image translation.

Interpolation	CHI							CNS
**Derivative**	**PS-DCT1**	**PS-DCT2**	**PS-DFT**	**1-FD**	**2-CLFD**	**4-CLFD**	**4-CTFD**	–
airplane	22.87	27.00	21.40	23.99	25.40	25.14	27.05	**30.77**
Barbara	26.05	**28.36**	19.95	18.18	18.21	20.54	23.74	24.68
boat	25.29	27.91	20.29	23.49	24.21	24.73	27.00	**29.82**
bridge	25.25	**27.08**	21.17	19.63	18.34	19.45	21.04	21.35
building	24.43	26.86	21.41	23.46	25.55	25.36	27.50	**32.70**
cameraman	25.68	**27.46**	20.31	21.20	21.26	22.30	24.22	25.40
girl	25.14	27.20	22.35	23.71	24.94	24.97	26.79	**29.81**
LAX	23.45	**22.55**	21.01	15.24	13.61	14.69	16.20	16.41
Lenna	25.31	27.56	21.74	23.19	24.71	25.15	27.35	**30.84**
lighthouse	25.03	**28.19**	21.28	22.36	22.50	23.37	25.28	26.63
text	25.72	28.49	21.45	22.52	23.95	25.01	27.19	**29.50**
woman	20.01	26.69	25.73	23.08	24.08	24.19	26.29	**29.31**
average	24.52	27.11	21.51	21.67	22.23	22.91	24.97	**27.27**

**Table 2:** SNRs (dB) of the final output after successive image translation (m=1).

Interpolation	CHI							CNS
**Derivative**	**PS-DCT1**	**PS-DCT2**	**PS-DFT**	**1-FD**	**2-CLFD**	**4-CLFD**	**4-CTFD**	–
airplane	27.46	**44.24**	34.28	27.16	27.74	29.57	31.86	31.98
Barbara	37.58	**42.89**	29.93	18.62	18.43	21.24	24.84	24.86
boat	35.18	**46.02**	31.21	25.56	25.51	27.58	30.48	30.56
bridge	28.49	**32.42**	28.76	20.04	18.52	19.82	21.44	21.44
building	39.77	**44.92**	33.99	26.45	28.28	31.09	34.54	34.85
cameraman	36.95	**38.01**	30.47	22.20	21.80	23.58	25.58	25.60
girl	38.43	**42.45**	35.13	26.27	26.75	28.52	30.57	30.66
LAX	**28.60**	28.19	27.72	15.52	13.73	14.94	16.46	16.46
Lenna	40.30	**44.73**	33.60	25.27	26.31	28.80	31.67	31.79
lighthouse	30.84	**39.16**	31.48	23.59	23.17	24.88	26.87	26.90
text	33.58	**42.63**	31.88	23.79	24.95	27.42	30.01	30.08
woman	22.30	**41.11**	42.62	25.74	25.83	27.61	30.15	30.24
average	33.29	**40.57**	32.59	23.35	23.42	25.42	27.87	27.95

**Table 3:** SNRs (dB) of the final output after successive image rotation.

Interpolation	CHI							CNS
**Derivative**	**PS-DCT1**	**PS-DCT2**	**PS-DFT**	**1-FD**	**2-CLFD**	**4-CLFD**	**4-CTFD**	–
airplane	30.15	**31.66**	30.85	25.54	25.51	27.16	28.69	24.83
Barbara	27.14	**27.82**	27.41	18.80	18.65	20.55	22.86	19.16
boat	31.42	**33.55**	32.37	26.71	26.69	28.30	30.07	25.60
bridge	22.36	**22.58**	22.46	18.21	18.14	19.07	20.01	18.33
building	30.02	**31.86**	30.68	24.67	24.64	26.73	28.58	24.14
cameraman	26.83	**27.52**	27.14	21.29	21.25	22.76	24.16	21.42
girl	29.27	**30.34**	29.43	24.79	24.77	26.41	27.77	24.60
LAX	18.42	**18.49**	18.46	14.47	14.42	15.20	16.02	14.73
Lenna	30.30	**31.79**	30.90	24.91	24.88	26.70	28.49	24.50
lighthouse	24.55	**24.95**	24.72	19.60	19.53	20.65	21.72	19.68
text	25.86	**26.49**	26.03	20.34	20.26	21.83	23.23	20.40
woman	30.93	**32.28**	31.44	26.46	26.46	27.99	29.43	25.90
average	27.27	**28.28**	27.66	22.15	22.10	23.61	25.09	21.94

**Table 4:** Arithmetic operations for PS-DCT1, PS-DCT2, and PS-DFT in real numbers.

Method	Multiplications	Additions
PS-DCT1	$\frac{N}{2}\left(3\log_{2}N-10\right)+4\log_{2}N+6+N$	$\frac{7}{2}N\left(\log_{2}N-2\right)+4\log_{2}N+6$
PS-DCT2	$2N(\frac{3}{4}\log_{2}N-1)+6+N$	$N(\frac{7}{2}\log_{2}N-4)+6$
PS-DFT	$3(N\log_{2}N+N)$	$4N\log_{2}N+3\left(N\log_{2}N+N\right)$

**Table 5:** Mean execution time (s) for successive image translation and successive image rotation. CHI, cubic Hermite interpolation.

Method	Translation	Rotation
CHI-PS-DCT1	0.058	0.436
CHI-PS-DCT2	0.052	0.417
CHI-PS-DFT	0.037	0.391
CHI-1-FD	0.019	0.357
CHI-2-CLFD	0.021	0.362
CHI-4-CLFD	0.024	0.372
CHI-4-CTFD	1.455	3.207
CNS	3.399	92.866
